# Variations in Greater Trochanter Height as a Relation to Patient Demographics: Implications for Femoral Nail Design

**DOI:** 10.1155/aort/3214878

**Published:** 2025-05-21

**Authors:** Jordan A. Haber, Amogh I. Iyer, Matthew Dulas, Douglas Weaver, Jason A. Strelzow

**Affiliations:** ^1^Department of Orthopaedic Surgery, University of Michigan, Ann Arbor, Michigan, USA; ^2^College of Medicine, Ohio State University, Columbus, Ohio, USA; ^3^Department of Orthopedics and Rehabilitation, University of Wisconsin, Madison, Wisconsin, USA; ^4^Department of Orthopaedic Surgery and Rehabilitation Medicine, University of Chicago, Chicago, Illinois, USA; ^5^Department of Orthopaedic Surgery, Washington University in St. Louis, St. Louis, Missouri, USA

**Keywords:** cephalomedullary lag screw, femoral fracture, implant, intramedullary nail fixation, tip-apex distance

## Abstract

Intramedullary nail fixation is the “gold standard” for surgical intervention of femoral fractures. While many aspects of nail design and patient anatomy have received specific focus, anatomic variations in proximal femoral geometry and greater trochanteric height variation have been poorly studied. Understanding the potential relationship of the greater trochanter to patient demographics may provide an opportunity to improve implant placement. Retrospective review of x-ray and computed tomography images of the proximal femur was performed. Inclusion criteria consisted of skeletally mature patients over 18 years old with imaging of the proximal femur. Inclusion criteria identified 296 patients. Mean age of included patients was 34 ± 20 years. Average greater trochanter height was 40 ± 8.1 mm. Mean caput-collum-diaphyseal angle was 141 ± 8.6 degrees. After identifying significant factors using univariate analyses a multivariable linear regression demonstrated that patient height and caput-collum-diaphyseal angle were statistically significant predictors for greater trochanter height. For every 1 cm increase in patient height there was a predicted 0.11 mm increase in greater trochanter height (*p*=0.01). Conversely, every 1 degree increase in caput-collum-diaphyseal angle results in an associated 0.17 mm decrease in greater trochanter height (*p* < 0.001). This study provides information that may allow for the potential optimization of implant design or implant position to minimize proximal nail protrusion, enhance nail fit and ensure cephalomedullary lag screw position in the head based on the proximal nail dimensions of the implant used.

## 1. Introduction

Intramedullary nail fixation is widely considered to be the “gold standard” of surgical intervention for femoral fractures [1]. Although no one implant provides a perfect device, improvements in nail design continue to help surgeons recreate patient anatomy and prevent implant related complications [2]. Recent nail design changes including altering nail radius of curvature to accommodate femoral bow and related differences with patient parameters are examples of implant improvements (Stryker Gamma 3, Synthes TNF-Alpha, and Zimmer Natural nail) [3–5]. Other aspects of the nail design, however, do not accommodate for potential patient specific variations, particularly given the paucity of literature evaluating patient specific anatomical variations in proximal femoral morphology [6, 7]. A lack of intramedullary nail variation, and patient-implant mismatch, may lead to higher rates of implant related irritation or complications including nail cut out, great trochanteric (GT) protrusion and wedge deformity [3]. One such concern is intramedullary nail protrusion from the GT into the surrounding tissue which has been associated with early implant failure, peri-implant fracture, and revision surgery [8].

Intramedullary nail protrusion occurs when the proximal nail length, typically the portion of the nail above the cephalomedullary screw, is greater than the height of the GT [9]. GT height is defined as the vertical distance between the point where the femoral neck axis and femoral shaft axis meet, and the tip of the GT [10]. Several existing parameters have been identified to optimize implant selection, including tip-apex distance (TAD) [11]. TAD refers to the sum of the distance from the tip of the lag screw to the apex of the femoral head on an anteroposterior radiograph and is independent of the GT [12]. Optimizing TAD while also attempting to reduce implant related complications including proximal nail protrusion can be challenging given the current nail designs with fixed proximal nail dimensions and no variations in length specific proximal morphology [13]. A TAD greater than 24 mm has been shown to be associated with higher rates of implant-related complications, such as cutout and implant failure [14, 15]. Imperfect positioning of either the lag screw in the femoral head or proximal protrusion of the IMN in the GT can require intraoperative surgical decision making to optimize screw position at the potential expensive of nail protrusion proximally. If the position of the proximal lag screw hole in the nail is too low, the surgeon may be required to sink the implant to improve the positioning of the lag screw. This may lead to lower quality proximal fixation due to loss of femoral cortical contact with the proximal nail but optimized TAD. Conversely, if the lag screw position is optimal but there is proximal nail protrusion from the GT, this may enhance femoral fixation while risking patient discomfort, implant prominence and IMN failure [16].

An understanding of the relationship of proximal femoral anatomy, particularly the height of the GT, may be used with other established fixation tools to optimize the fixation points during intramedullary nail fixation procedures. Such optimization may lead to lower rates of implant failure and postoperative complications while lessening difficult intraoperative decision-making. There has been previous work evaluating the effect of patient height and ethnicity on parameters of femoral shaft, noting that differences in the femoral bow among patients is largely due to variation in height and sex among different ethnicities, but no such evaluation has been performed for the GT [5–7]. Patient demographics such as age, sex, height, weight, BMI, or ethnicity of the patient may play an important role in these dimensions [6]. Early data currently exists to support a potential link with demographic and proximal femoral morphological variation. A 2021 study found that intramedullary nail cutout was significantly elevated in patients of Asian descent compared to Caucasian counterparts with the authors suggesting such failure may be linked to shorter stature, shorter GT heights and larger caput-collum-diaphyseal (CCD) angles [8]. Linkage of the GT to the femoral height on a patient-specific basis may allow prediction of lag screw position and an optimal IMN fixation technique, reducing the incidence of IMN failure and postoperative complications. The primary objective of this study was to determine if patient-specific demographics impact the proportion of the GT to the femur. The secondary objective was to determine if the ideal positioning of intramedullary nail fixation points differs across various patient demographics.

## 2. Methods

An urban level 1 trauma center patient database was utilized to identify patients with proximal femoral X-rays and CT scans between March 1^st^, 2020, and January 1^st^, 2021. This study is a level of evidence III. Approval was obtained from the institutional review board. This institutional database is comprehensive for orthopedic patients, providing a large population for consideration. A retrospective chart review was subsequently conducted. All images were evaluated through the electronic medical record (EMR) and picture archiving system (PACS).

**Figure 1:**
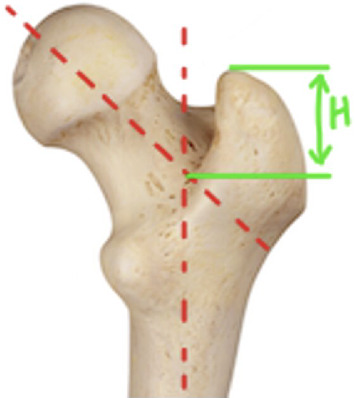
Measurement of the height of the greater trochanter, with variable “*H*” representing the height of the GT.

**Figure 2:**
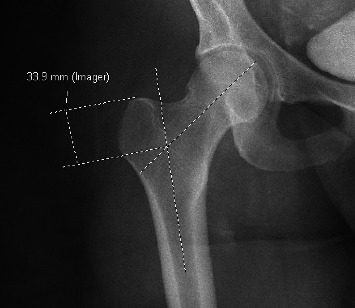
Measurement of GT height via x-ray image in EMR/PACS, with 33.9 mm representing the height of the GT.

**Figure 3:**
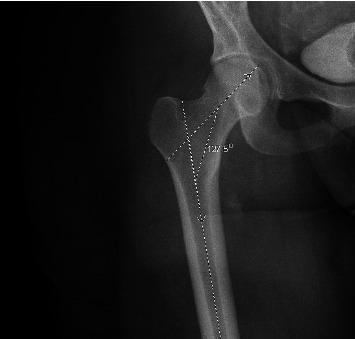
Measurement of CCD angle via x-ray image in EMR/PACS, with 127.5 degrees representing the CCD angle.

**Figure 4:**
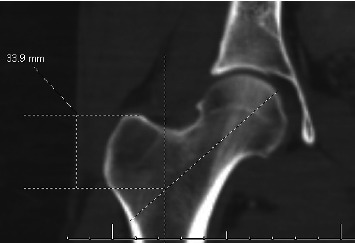
Measurement of GT height via CT image in EMR/PACS, with 33.9 mm representing the height of the GT.

**Figure 5:**
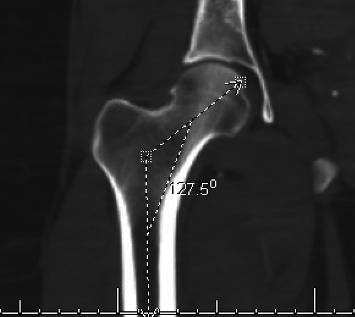
Measurement of CCD angle via CT image in EMR/PACS, with 127.5 degrees representing the CCD angle.

All patients underwent a standardized set of x-ray measurements including GT height and CCD angles. An additional measurement was the distance from the piriformis fossa to the tip of the GT which served as an internal control variable. This additional measure of GT height provided an internal control to account for potential variations in measurement, imaging rotation and patient morphological differences in femoral neck-shaft angle. Patient demographics were collected including age, sex, height, weight, BMI, and ethnicity. Exclusion criteria consisted of patients with a fractured or malunited proximal femur or GT from prior injury, and patients whose x-ray or CT scan was not obtained at the institution of the study. Measurements were made in EMR/PACS using online calibrated digital tools. To minimize variation, all measurements were obtained using the coronal images when the GT, femur, and femoral head were simultaneously in plane. Additionally, a single observer analyzed all images and performed bilateral measurements to ensure internal consistency. x-ray images of GT height measurement are demonstrated in Figures 1 and 2, while x-ray CCD angle is demonstrated in Figure 3. A CT image of GT height measurement is demonstrated in Figure 4, while x-ray CCD angle is demonstrated in Figure 5.

### 2.1. Statistical Methods

Quantitative statistical analysis was employed to analyze the data. Mean ± standard deviation was reported for all continuous variables. Continuous variables were compared using a 2-Sample *t*-Test. Average GT height, CCD angle and piriformis fossa to GT tip distance were averaged taking the left and right measurements. If only a unilateral measurement was available, that was taken as the value for the average calculation. Univariate linear regression analysis using age, sex, ethnicity, height, weight, BMI, CCD angle, and distance from the piriformis fossa to the GT tip as predictors for the outcome variable of GT height was conducted. Any predictor that was statistically significant in the univariate models was used to create a multivariate linear regression model. Statistical significance was determined at a threshold of *p* < 0.05, and all statistical analyses were carried out using two-sided tests. Statistical analyses were performed using R Statistical Software version 4.1.0.

## 3. Results

After database review, there were 365 patients considered for data analysis with 58 excluded for duplicate entries following clinical re-evaluation or secondary imaging. An additional nine entries were excluded due to fractures in the zone of interest, causing inability to attain accurate measurements of the GT. After exclusions, 296 patients were ultimately analyzed. Of the 296 patients included, 290 patients had complete datasets for age, sex, height, weight, and BMI. A value for height was not recorded in the EMRs of eight patients, preventing BMI calculation. Additionally, 87 patients did not have bilateral GT measurements due to unilateral hip imaging. In total, 203 patients had complete bilateral measurements recorded for all datapoints of interest.

The mean age of included patients was 34.9 ± 20 years with 190 male (64.9%) and 106 female patients (35.8%). Two patients identified as transgendered. Both patients were transgender females, assigned male at birth. The birth gender of these patients was recorded and utilized. The mean patient height was 169.6 ± 15.4 cm. There were 229 Black/African American patients, 45 White patients, and 22 patients who identified as another ethnicity. The average BMI of the patient cohort was 26.9 ± 7.4 kg/m^2^. Mean CCD angle was 141. 3 ± 8.6 degrees with no differences between the right and left side detected (*p*=0.50). Similarly, the mean height of the GT calculated from the piriformis fossa to GT was 23.8 ± 4.5 mm. No difference was detected between sides (*p*=0.60). The mean calculated height of the GT was 40.0 ± 8.1 mm without significant variation between the left and right sides (*p*=0.76). Complete demographic information can be found in Table 1.

In univariate linear regression analysis, each variable was individually used as a predictor variable for GT height. Age CCD angle, sex, height, weight, and BMI were all statistically significant predictors. The predicted change in GT height (mm) for a unit change in the predictor variable along with the 95% confidence interval (CI) can be seen in Table 2. These significant predictor variables were then all included in the multivariate analysis (Table 3).

In multivariate linear regression, CCD angle and height were statistically significant predictors for GT height. There was a predicted −0.17 (−0.27, −0.08) mm decrease in GT height for every 1 degree increase in CCD angle (*p* < 0.001). Height was a significant predictor variable with a 0.11 (0.03, 0.19) mm predicted increase for every 1 cm increase in patients height (*p*=0.01) (Table 3). The residual standard error (RSE) for this model was 0.15.

## 4. Discussion

IMN fixation remains the cornerstone of surgical management for femoral fractures due to its biomechanical advantages and proven clinical outcomes [2]. However, achieving optimal fixation without causing implant-related complications, such as protrusion from the GT, remains an unsolved challenge [3, 9]. This study addresses a critical gap in the literature by investigating the influence of patient-specific demographics on proximal femoral morphology and its implications for IMN fixation in femoral fractures. With IMN fixation being the gold standard in surgical management for such fractures, understanding the relationship between patient demographics and femoral morphology is paramount for optimizing implant performance and reducing postoperative complications [2]. By examining factors such as age, sex, height, weight, and BMI, this research discusses the relation between patient characteristics and implant selection, emphasizing the need for tailored surgical approaches to accommodate individual anatomical variations. The current study underscores the importance of integrating patient-specific considerations into orthopedic practice, building toward enhanced surgical outcomes and improved patient care. These results confirm previous literature suggesting that patient demographics, including age, sex, height, weight, and BMI, influence proximal femoral morphology [6, 9, 17].

Moreover, this study's findings may help during clinical decision-making in femoral IMN fixation procedures. By elucidating the correlation between patient demographics, GT height, and CCD angle, the treating surgeons can potentially anticipate potential challenges and tailor surgical strategies accordingly. The observed variations in GT height and CCD angle based on patient characteristics highlight the need for further exploration of more nuanced approaches in implant selection and surgical technique to minimize complications such as nail protrusion and inadequate fixation. This study offers insight for possible future optimization of femoral IMN fixation and advancing orthopedic surgical practice towards more personalized and effective patient care. Despite high rates of operation success, there are several pitfalls to femoral IMF. When comparing antegrade nail fixation of the proximal femur, previous literature has demonstrated increased incidence in proximal nail protrusion among Asian patients compared to Caucasian patients [9, 17]. Similar to the findings of the current study, Sarai et al. suggest that the increased incidence of postoperative adverse events was due to shorter stature, shorter GT height, and larger CCD angle in Asian populations [9]. Although the study did not include Asian patients, a significant relation between patient height, GT height, and CCD angle was noted. Aligning with the studies initial hypothesis, the results of this study suggest that GT height and CCD angle vary based on the height, weight, age, and sex of patients. This finding is likely due to increased femoral height among taller patients, and male patients being taller than female patients when considering demographical and anthropometric means.

The current study has several limitations. Although both x-ray and CT evaluations were utilized to minimize the potential confounders of image rotation and technique these are not standardized research scans and thus variation may be present in the image calibration. The lack of standardized imaging protocols may have introduced errors in the measurement and would be an excellent avenue for further study. The measurements of the study were conducted by a single reviewer. Although consistency was shown in measurements and based on contralateral comparison, the single reviewer model could have introduced measurement error and influenced results. Regarding the ethnicity of patients, patients were categorized as Black, White, or Other based on the demographics of patients seen at the urban level one center in this study. Additional demographic and ethnicity variation would further expand on the findings of the current study to increase the generalizability of the results. There are several other ethnicities that could be considered in future studies, such as Hispanic, Asian, and Multiracial. The results can only be correlated with the patient population examined. When considering patient sex, only considered biological males and biological females. Consideration of transgender patients would provide another measure, specifically when considering transgender patients taking gender-affirming hormones. It is possible that transgender females who are taking estrogen and transgender males who are taking testosterone may have different measurements due to hormonal influence. The limited data set may have influenced the results. Clinical execution of study results will require further analysis and ‘buy-in' from implant manufactures with a potential to generate specific proximal nail morphology based on nail lengths which make the clinical impact of this study dependent on implant manufacturers.

## 5. Conclusions

The results of this study suggest that patient demographics influence the GT height. In particular height, CCD angle, and distance from the piriformis to GT tip had a significant predictive relationship with GT height, with patient height having a positive linear relationship and CCD angle having a negative linear relationship. Future studies should consider the design and fit characteristics of IMN fixation to potentially improve implant design and fixation techniques.

## Figures and Tables

**Table 1:** Demographics of the patient cohort.

		Totals	Std	
Age (years)		34.99	20.10	
Sex				
Female		106		
Male		190		
Height (cm)		169.61	15.36	
Weight (kg)		77.80	26.16	
BMI (km/m^2^)		26.96	7.40	
Ethnicity				
Black		229		
White		45		
Other		22		

**Measurement variables**				
	**Left**	**Right**	**Average**	**p** **value**

CCD angle (degrees)	140.29 (9.06)	142.50 (9.74)	141.29 (8.60)	0.5
Distance from PF to GT (mm)	23.1 (4.47)	24.03 (5.07)	23.82 (4.52)	0.6
GT height (mm)	39.98 (9.13)	39.73 (8.32)	40.00 (8.10)	0.76

**Table 2:** Univariate linear regression.

Predictor variable	Predicted GT height (mm) change/unit change	95% CI	*p* value
Age (years)	0.08	0.03, 0.13	**< 0.001**
CCD angle (degrees)	−0.026	−0.36, −0.15	**< 0.001**
Sex			**< 0.001**
Female	—	—	
Male	4.1	2.2, 6.0	
Height (cm)	0.27	0.21, 0.32	**< 0.001**
Weight (kg)	0.12	0.09, 0.15	**< 0.001**
BMI (kg/m^2^)	0.19	0.06, 0.31	**0.004**
Ethnicity			
Black	—	—	
Other	0.74	−2.8, 4.3	0.684
White	0.68	−2.0, 3.4	0.623

*Note:* The bold values represent values of significance with *p* < 0.05.

Abbreviation: CI = confidence interval.

**Table 3:** Multivariate analysis.

Characteristic	Predicted change in GT height (mm) for unit change in predictor	95% CI	*p* value
Age (years)	0.01	−0.03, 0.05	0.612
CCD angle (degrees)	−0.17	−0.027, −0.08	< 0.001
Sex			0.903
Female	—	—	
Male	0.12	−1.9, 2.0	
Height (cm)	0.11	0.03, 0.19	**0.01**
Weight (kg)	0.02	0.01, 0.06	0.235

*Note:* The bold value represents the value of significance with *p* < 0.05.

Abbreviation: CI = confidence interval.

## Data Availability

Data supporting the findings of this study are available from the corresponding author upon reasonable request. Access to the data will be granted in compliance with institutional and journal policies.
